# Determining the Optimal Number of Wearing-Days Given a Fixed Number of Accelerometers in Population-Level Study

**DOI:** 10.2188/jea.JE20180095

**Published:** 2019-11-05

**Authors:** Paul H. Lee

**Affiliations:** School of Nursing, Hong Kong Polytechnic University, Hong Kong

**Keywords:** accelerometry, epidemiology, measurement, optimization, statistics

## Abstract

**Background:**

In research using accelerometers to measure physical activity, the number of accelerometers that can be utilized in a study and the study duration are both constrained. It means that increasing the number of accelerometer wearing days for all subjects leads to a decrease in the total number of participants the study can recruit. We used simulations to find the optimal combination of the number of wearing days and number of participant given a fixed number of accelerometer days.

**Methods:**

Two scenarios were studied here, including estimation of population physical activity level and the association between physical activity level and a health outcome. Another similar simulation was conducted by bootstrapping the National Health and Nutrition Examination Survey (NHANES) 2003–2006 accelerometer data (*n* = 4,069).

**Results:**

The simulation results of the first scenario showed that the error was minimized when the number of wearing days was 1 to 2. Simulation results of the second scenario showed that the optimal number of wearing days increased with the total number of accelerometer days and decreased with intra-class correlation (ICC).

**Conclusion:**

We developed a tool for researchers to determine the optimal combination of the number of the accelerometer wearing days and the total number of participants and showed that 1 to 2 accelerometer wearing days is optimal for estimation of population physical activity level.

## INTRODUCTION

The use of accelerometers is becoming more popular in population-level research as an objective measurement of physical activity and sedentary behavior because they have demonstrated high validity and reliability.^1^^,^^2^ In research using accelerometers to measure physical activity, one important aspect regarding study design is to determine the number of accelerometer wearing days (that is, the number of days each subject was required to wear the accelerometer).^3^ With an objective to estimate the habitual physical activity and sedentary behavior levels of a group of subjects, increasing the measurement timeframe can improve the estimation by reducing the day-to-day variability. Currently, there are no definite guidelines about the minimum number of required wearing days as it is determined by many factors, including the study population, how the accelerometer raw data are transformed and outputted, and how the physical activity and sedentary behaviors outcome data are analyzed.^3^ In physical activity literature, the number of accelerometer wearing days used ranged from a day^4^ to a year,^5^ and most studies collected accelerometer for 7 consecutive days^6^ due to the workday pattern.^7^ Building on this commonly-used 7-day wearing period, some researchers proposed measuring 1–2 days is enough to capture 80–90% of the 7-day variation.^8^^–^^11^

Assuming that the physical activity level of all subjects in the target population follow the same probability distribution, the estimation of habitual physical activity will only improve when we increase the number of accelerometer wearing days, since the within-subject reliability will only increase. Several studies suggested adopting the number of wearing days that yield a within-subject reliability of at least 0.8.^12^^,^^13^ However, these suggestions were based on the assumption that the study sample size is fixed, and the cutoff of 0.8 was arbitrary. Practically, the number of accelerometers that can be utilized in a study is constrained. For example, if we have only one accelerometer, we can choose to recruit two subjects to wear an accelerometer for *n* days, or to recruit one subject to wear it for 2*n* days. It means that decreasing the number of accelerometer wearing days for all subjects leads to an increase in the total number of participants the study can recruit. In other words, it is possible that reducing the number of wearing days that reduce the within-subject reliability maybe beneficial, since more subjects could be recruited. There is a need to develop a framework for determining the optimal combination of number of accelerometer wearing days and total number of participants that can achieve the highest accuracy in estimating the physical activity level. We performed several simulation studies to find the optimal combination of the number of wearing days and the number of participant given a fixed number of accelerometer days (defined as the accelerometer wearing days times the total number of participants). Two scenarios were studied here, including estimation of population physical activity level and estimation of the association between physical activity level and a health outcome. We performed our study in two simulated populations, one randomly drawn from a normal distribution and the other one bootstrapped from the National Health and Nutrition Examination Survey (NHANES) 2003–2006 accelerometer data that represented a positively-skewed distribution. To examine the case when the sampling is biased, we performed two simulations with the same setting as above but in which participants that are more active had a higher chance of being selected and the mean estimate was weighted appropriately. We made our R syntax available for researchers to perform simulations in their own settings.

## METHODS

The details of the simulation studies are outlined as follows. In this study, we assumed that the total number of accelerometer days is fixed (= *N*), which equaled the number of participants (*n*) times the number of days the participants are instructed to wear an accelerometer (*k*; *N* = *nk*). Two scenarios were simulated, one with the objective to estimate the population daily physical activity level measured using accelerometer count per minute (CPM; assume that mean equals 314 count/min and standard deviation equals 271 count/min to mimic the situation of NHANES 2003–2006 as a population,^14^ and the estimation method is outlined in the next paragraph), and the other one with the objective to estimate the odds ratios (ORs) of physical activity on a particular disease.

In both scenarios, to apply a constraint of CPM ≥0, we assume the natural log CPM of subject *i*, *i* = (1,…, *n*) at day *j*, *j* = (1,…, *k*) be *PA_ij_*. and *PA_ij_* equals μ + ε_γ,_*_i_* + ε_η,_*_i_*_,_*_j_*, where ε_γ,_*_i_* is the individual-level error and ε_η,_*_i_*_,_*_j_* is the day-to-day variability of subject *i*, and both individual-level and day-to-day random errors follow normal distribution with a mean of zero and variance of σ^2^ε_γ_ and σ^2^ε_η_, respectively. In the simulation, we assumed that the natural log CPM (= *PA_ij_*) of each subject was randomly drawn from a normal distribution with a mean of μ = 5.48 and a standard deviation of σ = 0.74, so that the mean and standard deviation of CPM equal 314 ($=exp^{\left(\mu +\sigma^{2}/2\right)}$) and 271 ($=\sqrt{\left[\text{exp}(\sigma^{2})-1]\text{exp}(2\mu +\sigma^{2}\right)}$), respectively. The day-to-day variability of the simulated subjects was quantified using intra-class correlation (ICC), which equals σ^2^ε_γ_/(σ^2^ε_γ_ + σ^2^ε_η_). Following the practice in the literature, the sample mean was used to estimate the population mean. In the second scenario, we generated each simulated subject’s diabetes status according to their generated physical activity level. We first examined the association between physical activity (where the daily CPM data would be averaged to represent the subject’s physical activity level and categorized into four levels using the NHANES sample quartiles [first quartile: CPM < 197.6; second quartile: 197.6–286.5; third quartile: 286.5–403.3; fourth quartile: >403.3], with the fourth quartile as the reference group, fixed in all simulations) and diabetes in the NHANES sample with logistic regression. Then, the diabetes status of each simulated subject were generated according to this regression (in which the regression slopes, β, for the first, second, and third quartiles were 2.79, 2.01, and 1.21, respectively). We simulated different settings (ICC = 0.2, 0.5, 0.8, *k* = 1–7, *N* = 1,000–10,000) to examine their association with the optimal *k*.

The mean and root-mean-squared error (RMSE) of the mean accelerometer count per minute ($\sqrt{\sum_{i=1}^{10000}{\left(CPM_{i,1}-314\right)}^{2}/10000}$ where *CPM_i_*_,l_ denoted the CPM estimated from the randomly-drawn dataset *i*.) and OR were used to assess the performance of different combinations of *n* and *k* given a fixed *N*.

Another similar simulation was conducted by bootstrapping the NHANES 2003–2006 accelerometer data for participants with 7 valid accelerometer days (*n* = 4,069), (details of the selection of this subset of data have been published elsewhere^14^), in which 462 of them (11.4%) had self-reported diabetes and the OR of low physical activity level was 2.3. NHANES 2003–2006 bootstrapping data were used to mimic the rightly-skewed distribution commonly observed in accelerometer data. The descriptive statistics of these accelerometer data can be found elsewhere.^6^^,^^14^ In sum, this gender-balanced sample (2,089 males, 51.3%) contained 1,274 children aged 0–19 years (31.3%), 1,477 adults aged 20–59 years (36.3%), and 1,318 older adults aged 60 years or older (32.3%). We bootstrapped the NHANES 2003–2006 data with 10,000 replications to create additional datasets with *k* = 1–7 and *N* = 1,000–10,000. The RMSE of the mean accelerometer count per minute of those with and without diabetes (the mean accelerometer counts per minute for those with and without diabetes equaled 202 and 332 count/min, respectively) and the OR of low physical activity level on diabetes were reported. For a particular combination of *k* and *N*, the RMSEs of those with and without diabetes were determined using the formulae $\sqrt{\sum_{i=1}^{10000}{\left(CPM_{i,1}-202\right)}^{2}/10000}$ and $\sqrt{\sum_{i=1}^{10000}{\left(CPM_{i,2}-332\right)}^{2}/10000}$, respectively, and the RMSEs of the log OR of low physical activity level on diabetes were determined using the formulae $\sqrt{\sum_{i=1}^{10000}{\left(logOR_{i,1}-2.79\right)}^{2}/10000}$, $\sqrt{\sum_{i=1}^{10000}{\left(logOR_{i,2}-2.01\right)}^{2}/10000}$, and $\sqrt{\sum_{i=1}^{10000}{\left(logOR_{i,3}-1.21\right)}^{2}/10000}$ where *CPM_i_*_,1_, *CPM_i_*_,2_, *OR_i_*_,1_, *OR_i_*_,2_, and *OR_i_*_,3_ represents the estimated mean accelerometer counts per minute of those with and without diabetes and the ORs of first, second, and third quartile of physical activity level on diabetes in the *i*-th simulation. The optimal value of *k* is the one that minimized the RMSEs. All RMSEs and ORs for one combination of *k* and *N* were based on 10,000 bootstrapped samples.

In addition, we created another sets of data with biasedness introduced in the aforementioned datasets by resampling the four quartiles of these datasets using the weighting of 0.8:1:1:1.2, so that the more active subjects were over-sampled in the resultant datasets. The RMSEs of the mean CPMs and ORs were weighted using the true weightings. All simulations were performed using R 3.3.0 (R Foundation for Statistical Computing, Vienna, Austria), and the syntax is available in eAppendix 1. For NHANES 2003–2006, consent was obtained from all participants, and the study was approved by the Centers for Disease Control ethics review board and has been performed in accordance with the ethical standards laid down in the 1964 Declaration of Helsinki.

In the following, we analytically determine the standard error for estimation of population physical activity level. We assume that all subjects have the same level of day-to-day variability). Under this assumption, the standard error of $\hat{PA_{ij}}$ (the sample mean of *PA_ij_*) equals $\surd [(k\sigma \varepsilon_{\gamma }{}^{2}+\sigma \varepsilon_{\eta }{}^{2})/N]$, which only depends on *k* given a fixed *N*, and the smallest standard error of *PA_ij_* is achieved when *k* = 1.

## RESULTS

**Figure 1.**
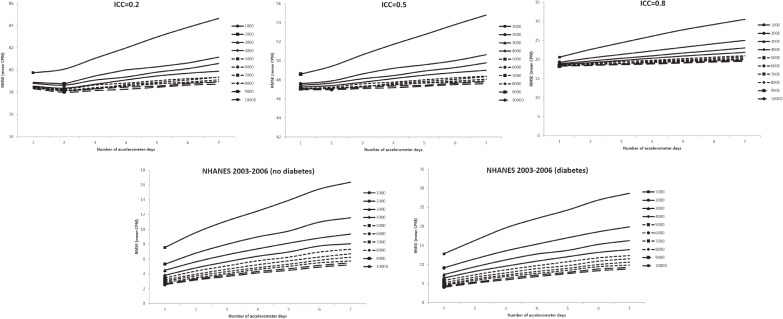
Simulation results with number of wearing days (mean count-per-minute) that achieved the minimum root mean squared error (RMSE) for given total accelerometer days marked with a symbol

**Figure 2.**
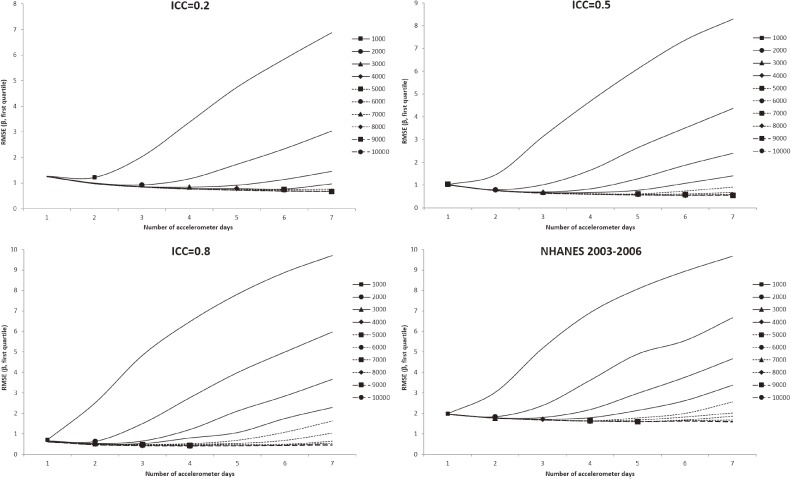
Simulation results with number of wearing days (regression slope of first quantile of physical activity on diabetes) that achieved the minimum root mean squared error (RMSE) for given total accelerometer days marked with a symbol

**Figure 3.**
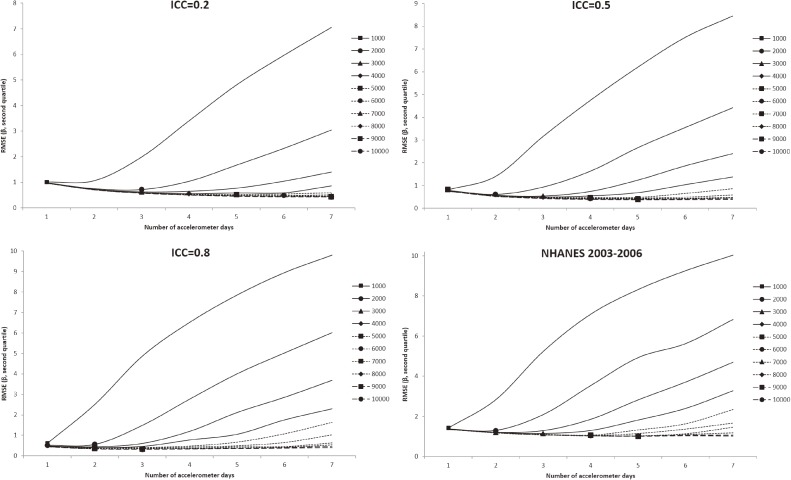
Simulation results with number of wearing days (regression slope of second quantile of physical activity on diabetes) that achieved the minimum root mean squared error (RMSE) for given total accelerometer days marked with a symbol

**Figure 4.**
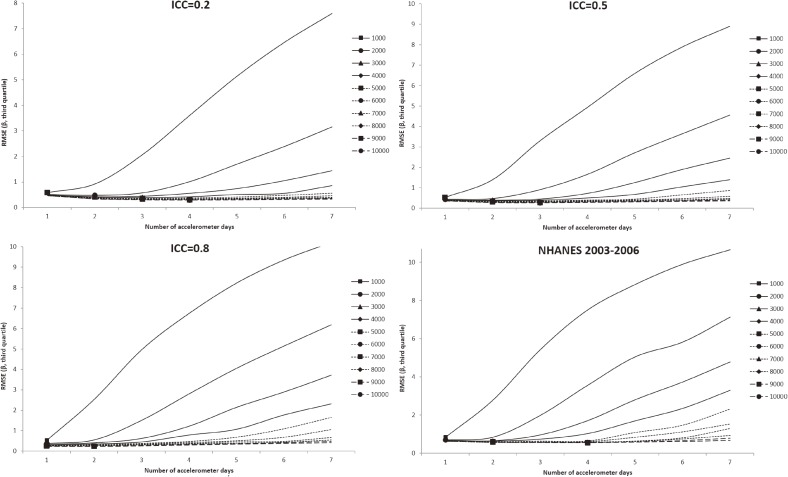
Simulation results with number of wearing days (regression slope of third quantile of physical activity on diabetes) that achieved the minimum root mean squared error (RMSE) for given total accelerometer days marked with a symbol

In all figures showing the simulation results, the optimal number of accelerometer days that yielded the smallest RMSE were marked with symbols. Figure 1 shows the RMSE of the mean accelerometer CPMs from the simulated data. For all ICCs (Figure 1, Figure 2, and Figure 3), wearing an accelerometer for 2 days are optimal, except for ICC of 0.2 and total number of accelerometer day of 1,000. Similarly, findings from the NHANES 2003–2006 bootstrapped datasets (with and without diabetes) showed that 1 single wearing day was preferred regardless of the number of participants and the total number of accelerometer day. Figure 2, Figure 3, and Figure 4 show the RMSE of the ORs from the simulated data. There are several general observations found from these results. First, the optimal number of wearing days increased with the total number of accelerometer days and decreased with ICC. Second, to estimate a stronger association (that is, the first quartile), wearing for more days (3 to 7) was preferred. Third, the optimal number of wearing days decrease with increasing ICC. For the NHANES bootstrapped data, the optimum number of accelerometer wearing days was similar to the case of ICC = 0.5.

Table 1, Table 2, and Table 3 show the estimated mean and ORs in the simulated datasets. Table 4 shows the estimated mean and ORs in the NHANES 2003–2006 bootstrapped datasets. There were structural bias in the mean estimation among the simulated datasets and biased in OR estimation in both simulated and NHANES bootstrapped datasets. The biasedness of both mean and OR estimation reduced when ICC increased. In the NHANES 2003–2006 bootstrapped datasets, the mean estimation was unbiased for all combinations of *n* and *k*. The biasedness of OR estimation was insensitive to the total number of accelerometer days, but increasing the number of wearing days improved the OR estimation for total number of accelerometer days larger than 3,000. However, for small sample size of total number of accelerometer days of 2,000 or less, fewer wearing days was preferred. Number of wearing days of 8 and beyond were not tested in the current simulation study, but it is likely that they are optimal for large sample size and small ICC according to the trend of the current simulation results.

**Table 1.** Estimation of mean and odds ratio (ICC = 0.2)

Number of wearing days	1	2	3	4	5	6	7
	Mean (true = 314.36)						

Total number of accelerometer days							

1,000	393.95	393.58	393.88	394.11	394.42	394.63	394.72
2,000	393.82	393.32	393.69	393.91	393.88	393.89	394.07
3,000	393.97	393.56	393.79	393.84	394.06	394.14	394.38
4,000	393.86	393.51	393.68	393.88	394.06	394.14	394.15
5,000	393.83	393.50	393.72	393.76	393.84	393.87	393.86
6,000	393.83	393.45	393.50	393.75	393.84	393.93	394.08
7,000	393.94	393.52	393.62	393.72	393.82	393.87	393.98
8,000	393.91	393.56	393.73	393.80	393.93	393.88	393.92
9,000	393.94	393.56	393.65	393.73	393.75	393.89	393.94
10,000	393.85	393.47	393.54	393.58	393.69	393.81	393.83

	β (first quartile, true = 2.79)						

Total number of accelerometer days							

1,000	1.56	1.91	2.26	2.81	3.54	4.29	5.11
2,000	1.55	1.85	2.01	2.16	2.33	2.54	2.80
3,000	1.54	1.84	2.00	2.10	2.19	2.27	2.36
4,000	1.54	1.83	1.98	2.08	2.15	2.20	2.27
5,000	1.53	1.82	1.97	2.07	2.13	2.19	2.24
6,000	1.53	1.82	1.97	2.07	2.13	2.18	2.22
7,000	1.54	1.82	1.97	2.06	2.12	2.18	2.21
8,000	1.54	1.82	1.97	2.06	2.12	2.17	2.21
9,000	1.54	1.82	1.97	2.06	2.12	2.17	2.20
10,000	1.53	1.82	1.96	2.05	2.11	2.16	2.19

	β (second quartile, true = 2.01)						

Total number of accelerometer days							

1,000	1.07	1.40	1.74	2.30	3.01	3.75	4.53
2,000	1.06	1.35	1.51	1.66	1.85	2.05	2.32
3,000	1.06	1.34	1.50	1.61	1.70	1.79	1.88
4,000	1.06	1.33	1.49	1.59	1.66	1.72	1.79
5,000	1.06	1.33	1.48	1.58	1.65	1.71	1.76
6,000	1.05	1.33	1.48	1.58	1.65	1.70	1.74
7,000	1.06	1.33	1.48	1.58	1.64	1.70	1.74
8,000	1.06	1.33	1.48	1.57	1.64	1.69	1.73
9,000	1.06	1.33	1.48	1.57	1.63	1.68	1.72
10,000	1.05	1.33	1.47	1.57	1.64	1.68	1.72

	β (third quartile, true = 1.12)						

Total number of accelerometer days							

1,000	0.68	0.89	1.14	1.58	2.15	2.69	3.29
2,000	0.68	0.84	0.93	1.04	1.19	1.36	1.59
3,000	0.68	0.84	0.93	0.99	1.05	1.11	1.18
4,000	0.68	0.83	0.92	0.97	1.01	1.05	1.10
5,000	0.68	0.83	0.91	0.97	1.01	1.04	1.07
6,000	0.67	0.82	0.91	0.97	1.01	1.03	1.06
7,000	0.68	0.83	0.91	0.96	1.00	1.03	1.06
8,000	0.68	0.83	0.91	0.96	1.00	1.03	1.06
9,000	0.68	0.83	0.91	0.96	0.99	1.02	1.05
10,000	0.68	0.83	0.91	0.96	1.00	1.02	1.04

ICC, intra-class correlation.

**Table 2.** Estimation of mean and odds ratio (ICC = 0.5)

Number of wearing days	1	2	3	4	5	6	7
	Mean (true = 314.36)						

Total number of accelerometer days							

1,000	362.65	362.54	362.84	363.02	363.13	363.25	363.41
2,000	362.45	362.24	362.52	362.65	362.60	362.58	362.72
3,000	362.57	362.45	362.60	362.61	362.78	362.83	363.03
4,000	362.50	362.42	362.53	362.67	362.78	362.83	362.82
5,000	362.45	362.39	362.53	362.52	362.57	362.58	362.56
6,000	362.46	362.35	362.35	362.53	362.57	362.64	362.75
7,000	362.56	362.43	362.48	362.53	362.58	362.60	362.67
8,000	362.53	362.47	362.56	362.58	362.66	362.60	362.62
9,000	362.55	362.44	362.48	362.51	362.50	362.60	362.63
10,000	362.48	362.39	362.40	362.39	362.45	362.53	362.53

	β (first quartile, true = 2.79)						

Total number of accelerometer days							

1,000	1.82	2.23	2.86	3.68	4.66	5.72	6.61
2,000	1.81	2.10	2.27	2.47	2.79	3.16	3.59
3,000	1.80	2.08	2.22	2.33	2.44	2.60	2.77
4,000	1.80	2.07	2.20	2.29	2.36	2.44	2.52
5,000	1.79	2.06	2.19	2.27	2.33	2.39	2.44
6,000	1.79	2.06	2.19	2.27	2.32	2.37	2.40
7,000	1.79	2.06	2.18	2.26	2.32	2.36	2.39
8,000	1.79	2.06	2.18	2.26	2.31	2.35	2.38
9,000	1.79	2.06	2.18	2.25	2.31	2.34	2.37
10,000	1.79	2.06	2.18	2.25	2.30	2.34	2.36

	β (second quartile, true = 2.01)						

Total number of accelerometer days							

1,000	1.29	1.67	2.29	3.11	4.07	5.10	5.95
2,000	1.27	1.55	1.72	1.91	2.24	2.60	3.03
3,000	1.27	1.54	1.67	1.77	1.89	2.04	2.20
4,000	1.27	1.52	1.65	1.74	1.81	1.89	1.97
5,000	1.27	1.52	1.65	1.72	1.78	1.84	1.89
6,000	1.26	1.52	1.64	1.72	1.77	1.81	1.85
7,000	1.27	1.52	1.64	1.72	1.77	1.81	1.84
8,000	1.26	1.52	1.64	1.71	1.76	1.80	1.82
9,000	1.26	1.51	1.63	1.71	1.75	1.79	1.82
10,000	1.26	1.51	1.63	1.70	1.76	1.79	1.81

	β (third quartile, true = 1.12)						

Total number of accelerometer days							

1,000	0.79	1.04	1.56	2.28	3.00	3.84	4.33
2,000	0.79	0.94	1.05	1.20	1.49	1.82	2.20
3,000	0.78	0.93	1.00	1.06	1.14	1.28	1.42
4,000	0.78	0.91	0.99	1.04	1.08	1.14	1.20
5,000	0.78	0.91	0.98	1.02	1.06	1.10	1.13
6,000	0.78	0.91	0.98	1.02	1.05	1.08	1.10
7,000	0.78	0.91	0.97	1.01	1.05	1.07	1.09
8,000	0.78	0.91	0.97	1.02	1.04	1.07	1.08
9,000	0.78	0.91	0.97	1.01	1.04	1.06	1.07
10,000	0.78	0.91	0.97	1.01	1.04	1.05	1.07

ICC, intra-class correlation.

**Table 3.** Estimation of mean and odds ratio (ICC = 0.8)

Number of wearing days	1	2	3	4	5	6	7
	Mean (true = 314.36)						

Total number of accelerometer days							

1,000	333.72	333.90	334.02	334.17	334.34	334.45	334.47
2,000	333.61	333.76	333.76	333.81	333.92	334.04	334.05
3,000	333.64	333.69	333.79	333.87	333.95	333.93	333.98
4,000	333.66	333.61	333.66	333.68	333.72	333.77	333.83
5,000	333.59	333.62	333.66	333.69	333.71	333.69	333.68
6,000	333.66	333.64	333.71	333.72	333.73	333.77	333.76
7,000	333.70	333.66	333.69	333.72	333.76	333.75	333.78
8,000	333.61	333.60	333.57	333.60	333.58	333.61	333.62
9,000	333.61	333.63	333.67	333.66	333.67	333.68	333.75
10,000	333.58	333.55	333.58	333.58	333.59	333.62	333.62

	β (first quartile, true = 2.79)						

Total number of accelerometer days							

1,000	2.26	2.84	3.96	5.12	6.30	7.40	8.35
2,000	2.21	2.41	2.62	3.02	3.55	4.12	4.79
3,000	2.21	2.40	2.51	2.64	2.86	3.17	3.50
4,000	2.20	2.39	2.48	2.55	2.65	2.77	2.96
5,000	2.20	2.38	2.46	2.51	2.57	2.64	2.72
6,000	2.19	2.37	2.45	2.51	2.55	2.59	2.65
7,000	2.19	2.37	2.45	2.49	2.54	2.57	2.60
8,000	2.20	2.37	2.45	2.50	2.53	2.56	2.59
9,000	2.19	2.37	2.44	2.49	2.53	2.55	2.58
10,000	2.19	2.37	2.44	2.49	2.52	2.55	2.57

	β (second quartile, true = 2.01)						

Total number of accelerometer days							

1,000	1.64	2.20	3.31	4.44	5.61	6.66	7.55
2,000	1.59	1.78	1.98	2.36	2.89	3.44	4.10
3,000	1.60	1.77	1.88	2.00	2.21	2.51	2.84
4,000	1.59	1.76	1.84	1.91	1.99	2.11	2.30
5,000	1.59	1.76	1.83	1.87	1.92	1.98	2.05
6,000	1.59	1.75	1.82	1.86	1.90	1.93	1.99
7,000	1.59	1.74	1.81	1.85	1.88	1.91	1.94
8,000	1.59	1.75	1.81	1.85	1.88	1.91	1.93
9,000	1.59	1.75	1.81	1.85	1.88	1.90	1.92
10,000	1.59	1.74	1.81	1.84	1.87	1.89	1.91

	β (third quartile, true = 1.12)						

Total number of accelerometer days							

1,000	0.97	1.43	2.45	3.40	4.25	4.97	5.47
2,000	0.93	1.02	1.18	1.54	2.04	2.53	3.13
3,000	0.93	1.03	1.10	1.19	1.39	1.68	1.98
4,000	0.93	1.02	1.06	1.11	1.19	1.29	1.47
5,000	0.93	1.01	1.05	1.07	1.11	1.16	1.23
6,000	0.92	1.00	1.04	1.07	1.10	1.12	1.17
7,000	0.92	1.00	1.04	1.06	1.08	1.10	1.12
8,000	0.92	1.00	1.04	1.06	1.08	1.10	1.11
9,000	0.92	1.01	1.04	1.06	1.08	1.09	1.11
10,000	0.92	1.00	1.04	1.06	1.07	1.08	1.10

ICC, intra-class correlation.

**Table 4.** Estimation of mean and odds ratio (NHANES)

Number of wearing days	1	2	3	4	5	6	7
	Mean (no diabetes, true = 331.62)						

Total number of accelerometer days							

1,000	332.75	332.58	332.56	332.57	332.72	332.62	332.62
2,000	332.54	332.66	332.55	332.64	332.84	332.63	332.53
3,000	332.57	332.66	332.58	332.67	332.62	332.61	332.70
4,000	332.61	332.52	332.58	332.54	332.66	332.68	332.61
5,000	332.58	332.57	332.63	332.57	332.50	332.58	332.57
6,000	332.61	332.61	332.62	332.57	332.62	332.58	332.71
7,000	332.68	332.63	332.60	332.55	332.65	332.56	332.56
8,000	332.62	332.58	332.62	332.55	332.48	332.70	332.61
9,000	332.56	332.62	332.59	332.56	332.54	332.57	332.49
10,000	332.62	332.63	332.59	332.60	332.61	332.62	332.57

	Mean (diabetes, true = 201.98)						

Total number of accelerometer days							

1,000	202.12	202.04	202.03	202.22	202.02	201.72	202.01
2,000	201.85	202.08	201.89	201.98	201.83	201.99	201.94
3,000	202.08	202.06	201.93	201.95	201.88	201.68	202.02
4,000	202.02	202.01	201.93	201.89	201.99	202.00	202.12
5,000	201.94	201.96	202.01	201.84	202.01	201.82	201.80
6,000	201.92	201.94	201.97	201.92	201.97	202.02	202.02
7,000	202.04	201.93	201.91	201.95	201.95	202.13	201.79
8,000	202.07	201.94	202.01	202.03	201.95	201.83	201.83
9,000	201.94	202.01	202.12	201.97	201.86	201.98	201.95
10,000	202.00	201.99	201.96	201.93	201.87	201.96	201.89

	β (first quartile, true = 2.79)						

Total number of accelerometer days							

1,000	0.89	1.56	2.96	4.53	5.82	6.89	7.91
2,000	0.86	1.09	1.43	2.04	2.86	3.33	4.38
3,000	0.85	1.06	1.22	1.41	1.78	2.15	2.76
4,000	0.85	1.04	1.18	1.28	1.46	1.60	2.01
5,000	0.85	1.04	1.16	1.23	1.33	1.39	1.67
6,000	0.85	1.04	1.15	1.23	1.29	1.32	1.47
7,000	0.84	1.04	1.15	1.21	1.27	1.26	1.42
8,000	0.84	1.04	1.14	1.20	1.26	1.27	1.36
9,000	0.84	1.03	1.13	1.20	1.25	1.24	1.34
10,000	0.84	1.03	1.14	1.20	1.25	1.24	1.32

	β (second quartile, true = 2.01)						

Total number of accelerometer days							

1,000	0.72	1.37	2.78	4.35	5.61	6.59	7.51
2,000	0.69	0.91	1.26	1.88	2.69	3.16	4.22
3,000	0.68	0.88	1.05	1.25	1.62	1.98	2.60
4,000	0.68	0.87	1.01	1.12	1.30	1.44	1.86
5,000	0.68	0.87	0.99	1.07	1.17	1.23	1.52
6,000	0.67	0.86	0.99	1.07	1.13	1.16	1.32
7,000	0.67	0.86	0.99	1.05	1.11	1.11	1.27
8,000	0.67	0.86	0.98	1.05	1.10	1.11	1.22
9,000	0.67	0.86	0.97	1.04	1.09	1.08	1.19
10,000	0.67	0.85	0.97	1.04	1.08	1.08	1.17

	β (third quartile, true = 1.12)						

Total number of accelerometer days							

1,000	0.53	1.09	2.32	3.64	4.57	5.15	5.55
2,000	0.51	0.64	0.90	1.49	2.23	2.67	3.57
3,000	0.51	0.62	0.71	0.87	1.20	1.56	2.12
4,000	0.50	0.60	0.67	0.76	0.90	1.03	1.40
5,000	0.51	0.61	0.66	0.71	0.77	0.83	1.07
6,000	0.50	0.61	0.65	0.70	0.73	0.77	0.87
7,000	0.50	0.60	0.65	0.69	0.71	0.71	0.83
8,000	0.50	0.60	0.64	0.69	0.71	0.71	0.77
9,000	0.50	0.60	0.64	0.69	0.70	0.69	0.75
10,000	0.50	0.60	0.64	0.69	0.69	0.70	0.73

NHANES, National Health and Nutrition Examination Survey.

**Figure 5.**
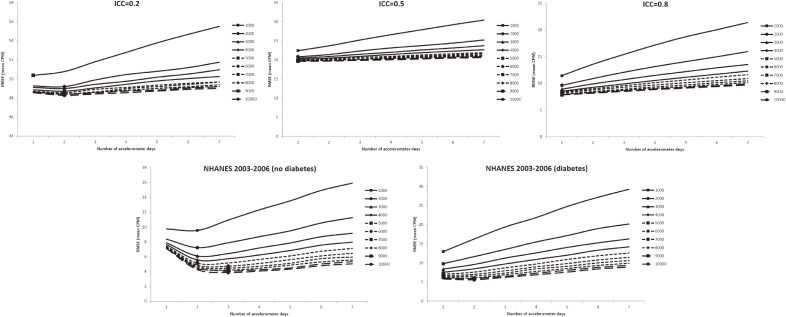
Simulation results (biased sample) with number of wearing days (mean count-per-minute) that achieved the minimum root mean squared error (RMSE) for given total accelerometer days marked with a symbol

**Figure 6.**
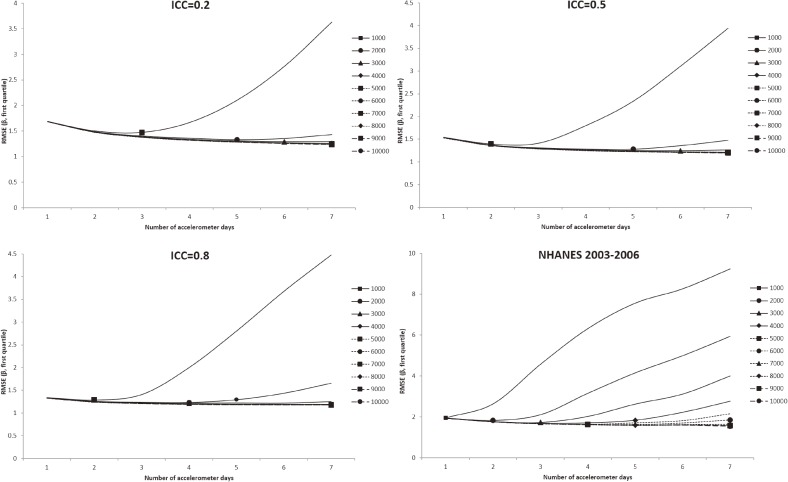
Simulation results (biased sample) with number of wearing days (regression slope of first quantile of physical activity on diabetes) that achieved the minimum root mean squared error (RMSE) for given total accelerometer days marked with a symbol

**Figure 7.**
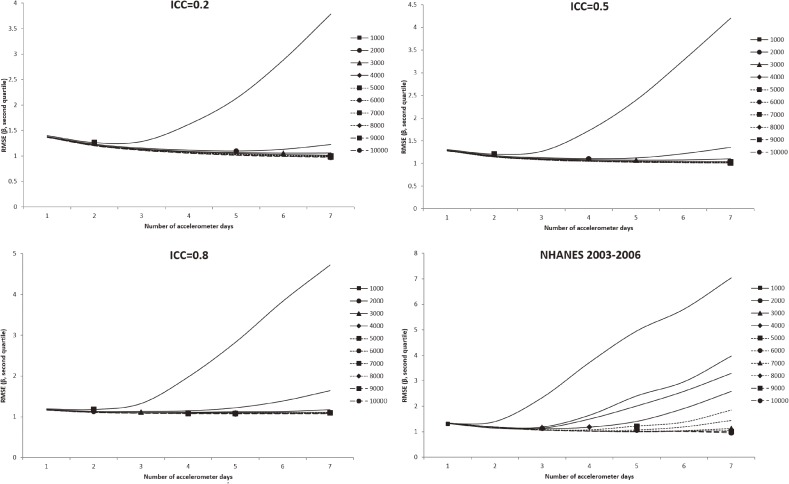
Simulation results (biased sample) with number of wearing days (regression slope of second quantile of physical activity on diabetes) that achieved the minimum root mean squared error (RMSE) for given total accelerometer days marked with a symbol

**Figure 8.**
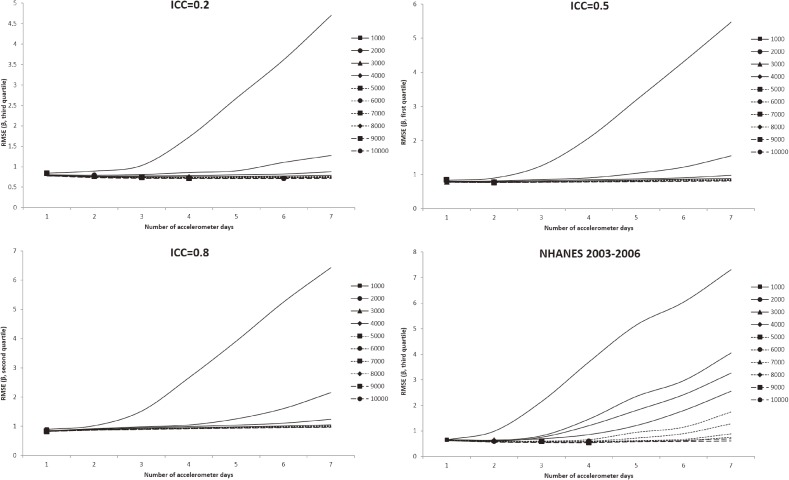
Simulation results (biased sample) with number of wearing days (regression slope of third quantile of physical activity on diabetes) that achieved the minimum root mean squared error (RMSE) for given total accelerometer days marked with a symbol

Figure 5 shows the RMSE of the mean accelerometer CPMs from the simulated data with a biased sample and weighted appropriately. For ICC of 0.2, 0.5, 0.8, and NHANES bootstrapped datasets of participants with diabetes, the results were similar with that of unbiased sample. For NHANES bootstrapped datasets of participants without diabetes, 2 and 3 accelerometer wearing days were optimal for total number of accelerometer day of 1,000–5,000 and 6,000–10,000, respectively. Figure 6, Figure 7, and Figure 8 show the RMSE of the ORs from the simulated data with a biased sample and weighted appropriately. Compare with an unbiased sample, more wearing days were preferred in a biased sample. For a small ICC of 0.2 and a medium ICC of 0.5, in estimating the effect of first and second quartile of physical activity on diabetes, 7 wearing days was optimal for a total number of accelerometer day of >4,000.

Table 5, Table 6, Table 7, and Table 8 show the estimated mean and ORs with a biased sample. It appeared that the biasedness in mean CPM was smaller than those in the unbiased sample (Table 1, Table 2, Table 3, and Table 4). In the NHANES 2003–2006 bootstrapped datasets, the biasedness in mean estimation decrease with the number of wearing days, and 4 to 7 days yielded similar performance. Similar to the simulation results in the unbiased sample, the biasedness of OR estimation was insensitive to the total number of accelerometer days, but increasing the number of wearing days improved the OR estimation for total number of accelerometer days larger than 3,000.

**Table 5.** Estimation of mean and odds ratio (ICC = 0.2, biased sample)

Number of wearing days	1	2	3	4	5	6	7
	Mean (true = 314.36)						

Total number of accelerometer days							

1,000	364.14	363.82	364.11	364.32	364.61	364.80	364.89
2,000	363.99	363.53	363.88	364.07	364.07	364.08	364.24
3,000	364.13	363.75	363.95	364.02	364.20	364.28	364.49
4,000	364.04	363.72	363.87	364.04	364.21	364.28	364.29
5,000	364.00	363.69	363.88	363.92	364.01	364.04	364.03
6,000	363.99	363.65	363.70	363.92	364.01	364.10	364.23
7,000	364.10	363.71	363.79	363.88	363.97	364.02	364.12
8,000	364.06	363.73	363.88	363.95	364.06	364.03	364.07
9,000	364.09	363.74	363.82	363.91	363.93	364.06	364.10
10,000	364.02	363.67	363.73	363.78	363.88	363.98	364.00

	β (first quartile, true = 2.79)						

Total number of accelerometer days							

1,000	1.13	1.35	1.47	1.59	1.73	1.88	2.15
2,000	1.12	1.33	1.44	1.50	1.55	1.59	1.62
3,000	1.11	1.33	1.43	1.49	1.54	1.57	1.59
4,000	1.11	1.33	1.43	1.49	1.53	1.57	1.59
5,000	1.11	1.32	1.43	1.49	1.54	1.56	1.59
6,000	1.11	1.32	1.42	1.49	1.53	1.56	1.58
7,000	1.11	1.32	1.43	1.49	1.53	1.56	1.58
8,000	1.11	1.32	1.42	1.48	1.52	1.55	1.58
9,000	1.11	1.32	1.42	1.49	1.52	1.56	1.58
10,000	1.11	1.32	1.42	1.48	1.52	1.55	1.57

	β (second quartile, true = 2.01)						

Total number of accelerometer days							

1,000	0.65	0.83	0.94	1.04	1.17	1.31	1.53
2,000	0.65	0.82	0.92	0.99	1.04	1.07	1.11
3,000	0.65	0.82	0.92	0.98	1.02	1.05	1.08
4,000	0.65	0.82	0.92	0.98	1.02	1.06	1.08
5,000	0.65	0.82	0.92	0.98	1.03	1.06	1.08
6,000	0.65	0.82	0.92	0.98	1.02	1.05	1.07
7,000	0.65	0.82	0.92	0.98	1.02	1.05	1.08
8,000	0.65	0.82	0.91	0.98	1.02	1.05	1.07
9,000	0.65	0.82	0.92	0.98	1.02	1.05	1.07
10,000	0.65	0.82	0.91	0.98	1.02	1.05	1.07

	β (third quartile, true = 1.12)						

Total number of accelerometer days							

1,000	0.35	0.39	0.42	0.44	0.41	0.41	0.47
2,000	0.35	0.40	0.42	0.42	0.44	0.43	0.44
3,000	0.35	0.40	0.42	0.43	0.44	0.45	0.45
4,000	0.35	0.40	0.43	0.44	0.44	0.45	0.45
5,000	0.35	0.40	0.43	0.44	0.44	0.45	0.45
6,000	0.35	0.40	0.42	0.44	0.44	0.45	0.45
7,000	0.35	0.40	0.42	0.44	0.45	0.45	0.46
8,000	0.35	0.40	0.42	0.43	0.44	0.45	0.45
9,000	0.35	0.40	0.42	0.43	0.44	0.45	0.45
10,000	0.35	0.40	0.42	0.43	0.44	0.45	0.45

ICC, intra-class correlation.

**Table 6.** Estimation of mean and odds ratio (ICC = 0.5, biased sample)

Number of wearing days	1	2	3	4	5	6	7
	Mean (true = 314.36)						

Total number of accelerometer days							

1,000	335.19	335.10	335.37	335.52	335.62	335.72	335.89
2,000	335.00	334.81	335.07	335.19	335.15	335.15	335.27
3,000	335.11	335.00	335.12	335.16	335.30	335.34	335.51
4,000	335.05	334.98	335.08	335.20	335.30	335.34	335.34
5,000	334.99	334.94	335.06	335.05	335.10	335.11	335.10
6,000	335.00	334.90	334.91	335.07	335.11	335.17	335.27
7,000	335.09	334.97	335.01	335.05	335.10	335.12	335.19
8,000	335.06	334.99	335.08	335.09	335.16	335.12	335.15
9,000	335.08	334.98	335.02	335.06	335.05	335.13	335.16
10,000	335.03	334.94	334.96	334.96	335.01	335.07	335.07

	β (first quartile, true = 2.79)						

Total number of accelerometer days							

1,000	1.27	1.46	1.56	1.70	1.87	2.13	2.47
2,000	1.27	1.45	1.53	1.58	1.61	1.65	1.69
3,000	1.26	1.44	1.52	1.56	1.60	1.62	1.64
4,000	1.26	1.44	1.52	1.57	1.60	1.62	1.64
5,000	1.26	1.44	1.52	1.56	1.59	1.61	1.63
6,000	1.26	1.43	1.51	1.56	1.59	1.61	1.62
7,000	1.26	1.44	1.51	1.56	1.59	1.61	1.63
8,000	1.26	1.43	1.51	1.55	1.58	1.60	1.62
9,000	1.26	1.43	1.51	1.55	1.58	1.60	1.62
10,000	1.26	1.43	1.51	1.55	1.58	1.60	1.61

	β (second quartile, true = 2.01)						

Total number of accelerometer days							

1,000	0.75	0.90	0.99	1.12	1.25	1.48	1.76
2,000	0.74	0.89	0.96	1.01	1.03	1.07	1.10
3,000	0.74	0.89	0.95	0.99	1.02	1.04	1.06
4,000	0.74	0.88	0.95	1.00	1.02	1.04	1.06
5,000	0.74	0.88	0.95	0.99	1.02	1.04	1.05
6,000	0.74	0.88	0.95	0.99	1.01	1.03	1.04
7,000	0.74	0.88	0.95	0.99	1.02	1.03	1.05
8,000	0.74	0.88	0.95	0.99	1.01	1.03	1.04
9,000	0.74	0.88	0.95	0.99	1.01	1.03	1.04
10,000	0.74	0.88	0.95	0.99	1.01	1.03	1.04

	β (third quartile, true = 1.12)						

Total number of accelerometer days							

1,000	0.35	0.37	0.35	0.38	0.34	0.34	0.37
2,000	0.35	0.37	0.37	0.36	0.35	0.36	0.34
3,000	0.35	0.37	0.37	0.37	0.36	0.36	0.35
4,000	0.35	0.37	0.38	0.37	0.37	0.36	0.36
5,000	0.35	0.37	0.37	0.37	0.37	0.36	0.36
6,000	0.35	0.37	0.37	0.37	0.37	0.36	0.36
7,000	0.35	0.37	0.37	0.37	0.37	0.37	0.37
8,000	0.35	0.37	0.37	0.37	0.36	0.36	0.36
9,000	0.35	0.37	0.37	0.37	0.36	0.36	0.36
10,000	0.35	0.37	0.37	0.37	0.37	0.36	0.36

ICC, intra-class correlation.

**Table 7.** Estimation of mean and odds ratio (ICC = 0.8, biased sample)

Number of wearing days	1	2	3	4	5	6	7
	Mean (true = 314.36)						

Total number of accelerometer days							

1,000	308.44	308.62	308.73	308.87	309.04	309.15	309.19
2,000	308.36	308.48	308.49	308.54	308.64	308.75	308.76
3,000	308.37	308.42	308.50	308.58	308.65	308.63	308.69
4,000	308.38	308.34	308.39	308.40	308.44	308.48	308.53
5,000	308.32	308.35	308.39	308.42	308.44	308.44	308.42
6,000	308.38	308.35	308.42	308.43	308.45	308.48	308.47
7,000	308.41	308.37	308.40	308.42	308.47	308.46	308.49
8,000	308.34	308.34	308.32	308.35	308.33	308.35	308.36
9,000	308.35	308.36	308.40	308.39	308.40	308.41	308.47
10,000	308.31	308.29	308.31	308.32	308.33	308.36	308.36

	β (first quartile, true = 2.79)						

Total number of accelerometer days							

1,000	1.49	1.60	1.68	1.83	2.10	2.45	2.84
2,000	1.47	1.57	1.61	1.64	1.67	1.71	1.78
3,000	1.47	1.56	1.60	1.63	1.64	1.66	1.68
4,000	1.47	1.56	1.60	1.62	1.64	1.66	1.67
5,000	1.46	1.56	1.60	1.62	1.63	1.65	1.65
6,000	1.46	1.56	1.59	1.62	1.63	1.65	1.66
7,000	1.46	1.56	1.59	1.61	1.63	1.64	1.65
8,000	1.47	1.56	1.59	1.61	1.63	1.64	1.65
9,000	1.46	1.56	1.59	1.61	1.63	1.64	1.65
10,000	1.46	1.55	1.59	1.61	1.62	1.64	1.64

	β (second quartile, true = 2.01)						

Total number of accelerometer days							

1,000	0.87	0.95	1.01	1.15	1.40	1.70	2.02
2,000	0.85	0.92	0.95	0.97	0.99	1.02	1.08
3,000	0.86	0.93	0.95	0.97	0.98	0.99	1.01
4,000	0.85	0.92	0.95	0.96	0.98	0.98	0.99
5,000	0.85	0.92	0.95	0.96	0.97	0.98	0.98
6,000	0.85	0.92	0.95	0.96	0.97	0.97	0.98
7,000	0.85	0.92	0.94	0.96	0.96	0.97	0.98
8,000	0.85	0.92	0.94	0.96	0.96	0.97	0.98
9,000	0.85	0.92	0.94	0.96	0.97	0.97	0.98
10,000	0.85	0.92	0.95	0.96	0.96	0.97	0.97

	β (third quartile, true = 1.12)						

Total number of accelerometer days							

1,000	0.31	0.28	0.24	0.21	0.22	0.19	0.10
2,000	0.30	0.27	0.25	0.22	0.21	0.19	0.17
3,000	0.31	0.27	0.25	0.24	0.22	0.22	0.21
4,000	0.31	0.28	0.26	0.24	0.23	0.22	0.21
5,000	0.31	0.27	0.25	0.23	0.22	0.21	0.21
6,000	0.31	0.27	0.25	0.24	0.23	0.22	0.21
7,000	0.30	0.27	0.25	0.23	0.22	0.21	0.21
8,000	0.30	0.27	0.25	0.23	0.22	0.21	0.21
9,000	0.30	0.27	0.25	0.24	0.23	0.22	0.21
10,000	0.30	0.27	0.25	0.23	0.22	0.22	0.21

ICC, intra-class correlation.

**Table 8.** Estimation of mean and odds ratio (NHANES, biased sample)

Number of wearing days	1	2	3	4	5	6	7
	Mean (no diabetes, true = 201.98)						

Total number of accelerometer days							

1,000	197.79	199.88	200.60	201.00	201.30	201.59	202.64
2,000	197.83	199.79	200.38	201.17	201.14	201.71	201.92
3,000	197.68	199.69	200.47	200.76	201.15	201.20	201.58
4,000	197.76	199.68	200.42	200.90	201.09	201.17	201.47
5,000	197.82	199.69	200.48	200.79	201.00	200.94	201.53
6,000	197.71	199.62	200.42	200.85	201.09	201.04	201.48
7,000	197.76	199.72	200.34	200.83	201.11	201.08	201.45
8,000	197.69	199.81	200.40	200.78	200.87	200.94	201.40
9,000	197.66	199.72	200.50	201.00	201.15	201.02	201.34
10,000	197.71	199.72	200.45	200.77	201.14	200.93	201.35

	Mean (diabetes, true = 331.62)						

Total number of accelerometer days							

1,000	325.00	328.59	329.83	330.56	330.77	330.76	331.52
2,000	324.92	328.51	329.88	330.61	330.95	330.70	331.26
3,000	324.90	328.61	329.88	330.38	330.98	330.58	331.41
4,000	324.86	328.49	329.78	330.57	330.87	330.90	331.36
5,000	324.91	328.53	329.81	330.50	330.88	330.83	331.23
6,000	324.91	328.51	329.78	330.45	330.95	330.72	331.36
7,000	324.94	328.51	329.86	330.49	330.80	330.71	331.31
8,000	324.89	328.52	329.80	330.49	331.01	330.80	331.31
9,000	324.90	328.54	329.80	330.49	330.84	330.76	331.33
10,000	324.87	328.50	329.84	330.43	330.87	330.74	331.35

	β (first quartile, true = 2.79)						

Total number of accelerometer days							

1,000	1.56	1.91	2.26	2.81	3.54	4.29	5.11
2,000	1.55	1.85	2.01	2.16	2.33	2.54	2.80
3,000	1.54	1.84	2.00	2.10	2.19	2.27	2.36
4,000	1.54	1.83	1.98	2.08	2.15	2.20	2.27
5,000	1.53	1.82	1.97	2.07	2.13	2.19	2.24
6,000	1.53	1.82	1.97	2.07	2.13	2.18	2.22
7,000	1.54	1.82	1.97	2.06	2.12	2.18	2.21
8,000	1.54	1.82	1.97	2.06	2.12	2.17	2.21
9,000	1.54	1.82	1.97	2.06	2.12	2.17	2.20
10,000	1.53	1.82	1.96	2.05	2.11	2.16	2.19

	β (second quartile, true = 2.01)						

Total number of accelerometer days							

1,000	1.07	1.40	1.74	2.30	3.01	3.75	4.53
2,000	1.06	1.35	1.51	1.66	1.85	2.05	2.32
3,000	1.06	1.34	1.50	1.61	1.70	1.79	1.88
4,000	1.06	1.33	1.49	1.59	1.66	1.72	1.79
5,000	1.06	1.33	1.48	1.58	1.65	1.71	1.76
6,000	1.05	1.33	1.48	1.58	1.65	1.70	1.74
7,000	1.06	1.33	1.48	1.58	1.64	1.70	1.74
8,000	1.06	1.33	1.48	1.57	1.64	1.69	1.73
9,000	1.06	1.33	1.48	1.57	1.63	1.68	1.72
10,000	1.05	1.33	1.47	1.57	1.64	1.68	1.72

	β (third quartile, true = 1.12)						

Total number of accelerometer days							

1,000	0.68	0.89	1.14	1.58	2.15	2.69	3.29
2,000	0.68	0.84	0.93	1.04	1.19	1.36	1.59
3,000	0.68	0.84	0.93	0.99	1.05	1.11	1.18
4,000	0.68	0.83	0.92	0.97	1.01	1.05	1.10
5,000	0.68	0.83	0.91	0.97	1.01	1.04	1.07
6,000	0.67	0.82	0.91	0.97	1.01	1.03	1.06
7,000	0.68	0.83	0.91	0.96	1.00	1.03	1.06
8,000	0.68	0.83	0.91	0.96	1.00	1.03	1.06
9,000	0.68	0.83	0.91	0.96	0.99	1.02	1.05
10,000	0.68	0.83	0.91	0.96	1.00	1.02	1.04

NHANES, National Health and Nutrition Examination Survey.

## DISCUSSION

A single day of measurement period has been suggested to estimate the population average physical activity level based on expert opinion.^3^ Some researchers also suggested a single day of measurement period based on its strong correlation with physical activity level yielded from 7-day measurement period.^8^^–^^10^ Here, we showed using analytic analysis that this recommendation is appropriate under certain assumptions. Result of this analytic analysis align with our unpublished results of the simulation studies with normally-distributed CPM that 1 single accelerometer wearing day minimized the RMSE regardless of the number of participants and ICC (data not shown). However, when the underlying assumptions are violated, for example when physical activity level follows log-normal distribution, wearing more days may be preferred.

There existed other methods to determine the optimal combination of accelerometer wearing days and number of participants, such as the Generalizability (G) Theory.^15^ Note that G Theory decomposes the reliability of a measurement into different facets and can be used to evaluate the reliability of a measurement when the objective is to estimate the population mean. Our simulation framework can also be applied when the objective is to estimate a causal relationship. As shown from the results, the optimal combination can be different under different objectives. The same framework can be applied to other types of accelerometer data outcomes, such as the number of steps, sitting time, or sleeping parameters. The optimum combination of the number of wearing days and number of participant for estimation of the association with a health outcome can be obtained by substituting the appropriate parameters (ICC, total number of accelerometer days, standard deviation of the error, the effect size of physical activity, and disease prevalence) in our R syntax. Similarly, the same framework can also be applied to estimate the causal effect of a variable on accelerometer data outcomes.

In general, if the objective is to estimate the association between physical activity level and a health outcome, the optimal number of wearing days depends on the number of accelerometers. For example, if there are limited number of accelerometers, the optimal number of wearing days would be 1 or 2 so that the sample size can be maximized. If more accelerometers are available, wearing 5 or more days would be desirable. When the sampling is biased, the number of accelerometer wearing days should be increased. The same simulation framework can be generalized to other study designs (for example randomized controlled trial with physical activity as an outcome), or when the physical activity level is a secondary variable (for example, as a confounder measured with error^16^).

One major limitation is that we assume all accelerometer wearing days are valid. Practically, some wearing days are invalid due to inadequate wearing time, and for precise sample size calculation, the invalid proportion has to be considered. Several existing studies had provided the statistics for invalid proportion estimation.^17^^,^^18^ Another limitation is that we assume physical activity level is consistent across all measurement days. Although this assumption appeared to be valid in the NHANES 2003–2006 accelerometer data (partial eta-square of the CPM across 7 days equaled 0.026, corresponded to a small effect size), it may not be applicable in other populations. It is commonly observed in some populations that physical activity level differed across weekdays and weekends.^19^ Treating the weekday activity and weekend activity as two distinct sub-populations, collecting accelerometer data of 1 weekday plus 1 weekend day would be appropriate to estimate population physical activity level. In addition, we used the sample mean to estimate the population mean and used logistic regression to analyze the association between physical activity and diabetes. These approaches ignored the heterogeneous and complex nature of the NHANES sample. However, this study serves as an introduction to the framework to determine the best combination of the number of wearing days and the number of participant given a fixed number of accelerometer days. Instead of presenting the results of all possible modelling choices here, we only showcased the most commonly-used ones and interested readers can modify our R syntax to fit their specific purposes. Finally, we assumed that the intra-subject and inter-subject variabilities are independent. However, one may argue that some active people only engage in physical activity in some days of a week, so they should have a higher day-to-day variability than their inactive counterparts. This phenomenon has been demonstrated in the NHANES 2003–2006 accelerometer data, evidenced by the strong correlation between mean physical activity count and intra-subject variance of the subjects who provided 7 valid accelerometer day data (ρ = 0.66, *P* < 0.001). Such a correlation can be addressed into our simulation framework in a fairly straightforward manner. In our simulation studies, we assume that log(*PA_ij_*) equals μ + ε_γ,_*_i_* + ε_η,_*_i_*_,_*_j_* and the intra-subject error follows normal distribution with constant variance, ie, ε_η,_*_i_*_,_*_j_* ∼ *N*(0, σ*_n_*^2^). We can modify the assumption to ε_η,_*_i_*_,_*_j_* ∼ *N*(0, α + βε_γ,_*_i_*σ*_n_*^2^) where α and β are constants to allow correlation between physical activity level and intra-subject variance. In addition, we can also use variance stabilization transformation could be used to reduce the dependence and our results should still be applicable on the transformed data.

### Conclusion

We developed a tool for researchers to determine the optimal combination of the number of accelerometer wearing days and the total number of participants, and showed that 1 to 2 accelerometer wearing days is optimal for estimation of population physical activity level. To estimate the causal effect of physical activity, a single wearing day is optimal when the total number of accelerometer day is small and the within-subject correlation is high. The number of wearing days increases with the total number of accelerometer day and decreases with the within-subject correlation.

## APPENDIX A. SUPPLEMENTARY DATA

eAppendix 1. R syntax for the simulation studies
