# Supplementary material for: Determining the Optimal Number of Wearing-Days Given a Fixed Number of Accelerometers in Population-Level Study
Source: J Epidemiol. 2019 Nov 5;29(11):432–43. doi: 10.2188/jea.JE20180095 (PMC6776478; doi:10.2188/jea.JE20180095)
Supplement: Supplementary file 1 [file je-29-432-s001.pdf]

eAppendix 1. R syntax for simulations

```
library(weights) # load the "weights" package
library(boot) # load the "boot" package

sim_size <- 10000 # simulation size
sim_mean <- rep(0,sim_size)

sample_size <- 9000 # number of accelerometer days
wearing_day <- 7 # maximum number of wearing days evaluated

sim_rmse <- matrix(0,nrow=sim_size,ncol=wearing_day)
sim_mean <- matrix(0,nrow=sim_size,ncol=wearing_day)
## mean with maximum available days
sim_mean_limit <- matrix(0,nrow=sim_size,ncol=wearing_day)
sim_error <- matrix(0,nrow=sim_size,ncol=wearing_day)
true_mean <- 5.478054 # mean of the log(data)
sd_1 <- 0.7438219 # inter-subject sd
ICC <- 0.8 # ICC of the data
sd_2 <- sd_1 * ((1-ICC)/ICC)^0.5
###### logistic regression parameter estimations
lnOR_1 <- 2.788 # true effect of quartile 1
lnOR_2 <- 2.014 # true effect of quartile 2
lnOR_3 <- 1.121 # true effect of quartile 3
lnslope <- -3.909 # proportion of the event
```

```
Q1 <- exp(qnorm(0.25, mean=5.478054, sd=0.7438219)) # define the cutoff of quartile 1
Q2 <- exp(qnorm(0.5, mean=5.478054, sd=0.7438219)) # define the cutoff of quartile 2
Q3 <- exp(qnorm(0.75, mean=5.478054, sd=0.7438219)) # define the cutoff of quartile 3
sim_lnOR_1 <- matrix(0,nrow=sim_size,ncol=wearing_day)
sim_lnOR_2 <- matrix(0,nrow=sim_size,ncol=wearing_day)
sim_lnOR_3 <- matrix(0,nrow=sim_size,ncol=wearing_day)
sim_lnOR_1_sig <- matrix(0,nrow=sim_size,ncol=wearing_day)
sim_lnOR_2_sig <- matrix(0,nrow=sim_size,ncol=wearing_day)
sim_lnOR_3_sig <- matrix(0,nrow=sim_size,ncol=wearing_day)
###### logistic regression parameter estimations end

###### biased sample parameter estimations
sim_rmse_biased <- matrix(0,nrow=sim_size,ncol=wearing_day)
sim_mean_biased <- matrix(0,nrow=sim_size,ncol=wearing_day)
sim_mean_limit_biased <- matrix(0,nrow=sim_size,ncol=wearing_day)
sim_error_biased <- matrix(0,nrow=sim_size,ncol=wearing_day)
sim_lnOR_1_biased <- matrix(0,nrow=sim_size,ncol=wearing_day)
sim_lnOR_1_sig_biased <- matrix(0,nrow=sim_size,ncol=wearing_day)
sim_lnOR_2_biased <- matrix(0,nrow=sim_size,ncol=wearing_day)
sim_lnOR_2_sig_biased <- matrix(0,nrow=sim_size,ncol=wearing_day)
sim_lnOR_3_biased <- matrix(0,nrow=sim_size,ncol=wearing_day)
sim_lnOR_3_sig_biased <- matrix(0,nrow=sim_size,ncol=wearing_day)
###### biased sample parameter estimations end

for (i in 1:sim_size){
```

```
accel_true <- rep(0,sample_size)

accel_true <- true_mean + sd_1 * rnorm(sample_size)


## create biased true accelerometer data

accel_biased <- accel_true

for (k in 1:sample_size){

if(runif(1) < 0.2 && ((accel_true[k]-true_mean)/sd_1) < -0.67448975) accel_biased[k] <-
2*true_mean - accel_true[k]

}


accel_data <- matrix(0,nrow=sample_size,ncol=wearing_day)

accel_data_biased <- matrix(0,nrow=sample_size,ncol=wearing_day) # biased data


for (k in 1:wearing_day){

accel_data[,k] <- accel_true + sd_2 * rnorm(sample_size)

accel_data_biased[,k] <- accel_biased + sd_2 * rnorm(sample_size) # biased data

}


## take exponential of the generated data

accel_true <- exp(accel_true)

accel_data <- exp(accel_data)

accel_data_biased <- exp(accel_data_biased)
```

```
## summarizing accelerometer data with mean of 1,...7 days

accel_summary <- matrix(0,nrow=sample_size,ncol=wearing_day)


accel_summary[,1] <- accel_data[,1]

accel_summary[,2] <- (accel_data[,1]+accel_data[,2])/2

accel_summary[,3] <- (accel_data[,1]+accel_data[,2]+accel_data[,3])/3

accel_summary[,4] <- (accel_data[,1]+accel_data[,2]+accel_data[,3]+accel_data[,4])/4

accel_summary[,5] <-
(accel_data[,1]+accel_data[,2]+accel_data[,3]+accel_data[,4]+accel_data[,5])/5

accel_summary[,6] <-
(accel_data[,1]+accel_data[,2]+accel_data[,3]+accel_data[,4]+accel_data[,5]+accel_data[,6])/6

accel_summary[,7] <-
(accel_data[,1]+accel_data[,2]+accel_data[,3]+accel_data[,4]+accel_data[,5]+accel_data[,6]+acc
el_data[,7])/7


accel_summary_biased <- matrix(0,nrow=sample_size,ncol=wearing_day)


accel_summary_biased[,1] <- accel_data_biased[,1]

accel_summary_biased[,2] <- (accel_data_biased[,1]+accel_data_biased[,2])/2

accel_summary_biased[,3] <-
(accel_data_biased[,1]+accel_data_biased[,2]+accel_data_biased[,3])/3

accel_summary_biased[,4] <-
(accel_data_biased[,1]+accel_data_biased[,2]+accel_data_biased[,3]+accel_data_biased[,4])/4

accel_summary_biased[,5] <-
(accel_data_biased[,1]+accel_data_biased[,2]+accel_data_biased[,3]+accel_data_biased[,4]+acc
el_data_biased[,5])/5

accel_summary_biased[,6] <-
(accel_data_biased[,1]+accel_data_biased[,2]+accel_data_biased[,3]+accel_data_biased[,4]+acc
el_data_biased[,5]+accel_data_biased[,6])/6
```

```
accel_summary_biased[,7] <-
(accel_data_biased[,1]+accel_data_biased[,2]+accel_data_biased[,3]+accel_data_biased[,4]+accel_data_biased[,5]+accel_data_biased[,6]+accel_data_biased[,7])/7



## error correlation summarizing accelerometer data with mean of 1,...7 days

for (k in 1:wearing_day){

sim_error[i,k] <- cor(accel_true, accel_summary[,k])

sim_error_biased[i,k] <- cor(accel_biased, accel_summary_biased[,k])

}


## compute weights


weight <- rep(1,sample_size)


for (k in 1:sample_size){

if(((accel_biased[k]-true_mean)/sd_1) < -0.67448975) weight[k] <- 5/4

if(((accel_biased[k]-true_mean)/sd_1) > 0.67448975) weight[k] <- 5/6

}


## accuracy of activity level

for (k in 1:wearing_day){

sim_rmse[i,k] <- mean((accel_summary[,k]-accel_true)^2)^0.5

# lognormal distrubution mean

miu <- mean(log(accel_summary[,k]))

sigmasq <- mean((log(accel_summary[,k])-miu)^2)
```

```
sim_mean[i,k] <- exp(miu + sigmasq/2)

miu_limit <- mean(log(accel_summary[1:floor(sample_size/k),k]))

sigmasq_limit = mean((log(accel_summary[1:floor(sample_size/k),k])-miu)^2)

sim_mean_limit[i,k] <- exp(miu_limit + sigmasq_limit/2)

}


## effect on disease

disease <- rep(0,sample_size)

disease_biased <- rep(0,sample_size)


for (j in 1:sample_size){

disease[j] <- sample(0:1,1,rep=TRUE,prob=c(1-
inv.logit(slope+lnOR_1*ifelse(accel_true[j]<Q1,1,0)+lnOR_2*ifelse((accel_true[j]>Q1 &
accel_true[j]<Q2),1,0)+lnOR_3*ifelse((accel_true[j]>Q2 &
accel_true[j]<Q3),1,0)),inv.logit(slope+lnOR_1*ifelse(accel_true[j]<Q1,1,0)+lnOR_2*ifelse((ac
cel_true[j]>Q1 & accel_true[j]<Q2),1,0)+lnOR_3*ifelse((accel_true[j]>Q2 &
accel_true[j]<Q3),1,0))))

disease_biased[j] <- sample(0:1,1,rep=TRUE,prob=c(1-
inv.logit(slope+lnOR_1*ifelse(accel_biased[j]<Q1,1,0)+lnOR_2*ifelse((accel_biased[j]>Q1 &
accel_biased[j]<Q2),1,0)+lnOR_3*ifelse((accel_biased[j]>Q2 &
accel_biased[j]<Q3),1,0)),inv.logit(slope+lnOR_1*ifelse(accel_biased[j]<Q1,1,0)+lnOR_2*ifels
e((accel_biased[j]>Q1 & accel_biased[j]<Q2),1,0)+lnOR_3*ifelse((accel_biased[j]>Q2 &
accel_biased[j]<Q3),1,0))))

}


## categorize accel data in 4 groups

accel_summary_4_bin_1 <- matrix(0,nrow=sample_size,ncol=wearing_day)

accel_summary_4_bin_2 <- matrix(0,nrow=sample_size,ncol=wearing_day)

accel_summary_4_bin_3 <- matrix(0,nrow=sample_size,ncol=wearing_day)
```

```
accel_summary_4_bin_biased_1 <- matrix(0,nrow=sample_size,ncol=wearing_day)

accel_summary_4_bin_biased_2 <- matrix(0,nrow=sample_size,ncol=wearing_day)

accel_summary_4_bin_biased_3 <- matrix(0,nrow=sample_size,ncol=wearing_day)


accel_summary_4_bin_1[,k] <- ifelse((accel_summary[,k]<Q1),1,0)

accel_summary_4_bin_2[,k] <- ifelse((accel_summary[,k]>Q1 & accel_summary[,k]<Q2),1,0)

accel_summary_4_bin_3[,k] <- ifelse((accel_summary[,k]>Q2 & accel_summary[,k]<Q3),1,0)

accel_summary_4_bin_biased_1[,k] <- ifelse(accel_summary_biased[,k]<Q1,1,0)

accel_summary_4_bin_biased_2[,k] <- ifelse((accel_summary_biased[,k]>Q1 &
accel_summary_biased[,k]<Q2),1,0)

accel_summary_4_bin_biased_3[,k] <- ifelse((accel_summary_biased[,k]>Q2 &
accel_summary_biased[,k]<Q3),1,0)


## fit logistic regression

for (k in 1:wearing_day){

reg <-
glm(disease[1:floor(sample_size/k)]~accel_summary_4_bin_1[1:floor(sample_size/k),k]+accel_
summary_4_bin_2[1:floor(sample_size/k),k]+accel_summary_4_bin_3[1:floor(sample_size/k),k
], family=binomial(logit))

se <- vcov(reg)^0.5

sim_lnOR_1[i,k] <- (reg$coefficients[2]) #OR

sim_lnOR_1_se <- se[2,2] #sd of adjusted effect

sim_lnOR_1_p <- 2-2*pnorm(abs(reg$coefficients[2])/se[2,2]) #p-value of z-test

if (sim_lnOR_1_p < 0.05) sim_lnOR_1_sig[i,k] <- 1 #indicator of significant p-value of OR

sim_lnOR_2[i,k] <- (reg$coefficients[3]) #OR

sim_lnOR_2_se <- se[3,3] #sd of adjusted effect

sim_lnOR_2_p <- 2-2*pnorm(abs(reg$coefficients[3])/se[3,3]) #p-value of z-test
```

```
if (sim_lnOR_2_p < 0.05) sim_lnOR_2_sig[i,k] <- 1 #indicator of significant p-value of OR

sim_lnOR_3[i,k] <- (reg$coefficients[4]) #OR

sim_lnOR_3_se <- se[4,4] #sd of adjusted effect

sim_lnOR_3_p <- 2-2*pnorm(abs(reg$coefficients[4])/se[4,4]) #p-value of z-test

if (sim_lnOR_3_p < 0.05) sim_lnOR_3_sig[i,k] <- 1 #indicator of significant p-value of OR


reg <-
glm(disease[1:floor(sample_size/k)]~accel_summary_4_bin_biased_1[1:floor(sample_size/k),k]
+accel_summary_4_bin_biased_2[1:floor(sample_size/k),k]+accel_summary_4_bin_biased_3[1:
floor(sample_size/k),k], family=binomial(logit))

se <- vcov(reg)^0.5

sim_lnOR_1_biased[i,k] <- (reg$coefficients[2]) #OR

sim_lnOR_1_se_biased <- se[2,2] #sd of adjusted effect

sim_lnOR_1_p_biased <- 2-2*pnorm(abs(reg$coefficients[2])/se[2,2]) #p-value of z-test

if (sim_lnOR_1_p_biased < 0.05) sim_lnOR_1_sig_biased[i,k] <- 1 #indicator of significant p-
value of OR

sim_lnOR_2_biased[i,k] <- (reg$coefficients[3]) #OR

sim_lnOR_2_se_biased <- se[3,3] #sd of adjusted effect

sim_lnOR_2_p_biased <- 2-2*pnorm(abs(reg$coefficients[3])/se[3,3]) #p-value of z-test

if (sim_lnOR_2_p_biased < 0.05) sim_lnOR_2_sig_biased[i,k] <- 1 #indicator of significant p-
value of OR

sim_lnOR_3_biased[i,k] <- (reg$coefficients[4]) #OR

sim_lnOR_3_se_biased <- se[4,4] #sd of adjusted effect

sim_lnOR_3_p_biased <- 2-2*pnorm(abs(reg$coefficients[4])/se[4,4]) #p-value of z-test

if (sim_lnOR_3_p_biased < 0.05) sim_lnOR_3_sig_biased[i,k] <- 1 #indicator of significant p-
value of OR

}
```

```
}

# output
sim_output <- matrix(0,nrow=56,ncol=2)

sim_output[1,1] <- mean(((sim_mean_limit[,1]-
exp(true_mean+sd_1^2/2))^2)^0.5)*(sim_size)^0.5
sim_output[2,1] <- mean(((sim_mean_limit[,2]-
exp(true_mean+sd_1^2/2))^2)^0.5)*(sim_size)^0.5
sim_output[3,1] <- mean(((sim_mean_limit[,3]-
exp(true_mean+sd_1^2/2))^2)^0.5)*(sim_size)^0.5
sim_output[4,1] <- mean(((sim_mean_limit[,4]-
exp(true_mean+sd_1^2/2))^2)^0.5)*(sim_size)^0.5
sim_output[5,1] <- mean(((sim_mean_limit[,5]-
exp(true_mean+sd_1^2/2))^2)^0.5)*(sim_size)^0.5
sim_output[6,1] <- mean(((sim_mean_limit[,6]-
exp(true_mean+sd_1^2/2))^2)^0.5)*(sim_size)^0.5
sim_output[7,1] <- mean(((sim_mean_limit[,7]-
exp(true_mean+sd_1^2/2))^2)^0.5)*(sim_size)^0.5

sim_output[8,1] <- mean(((sim_lnOR_1[,1]-lnOR_1)^2)^0.5)*(sim_size)^0.5
sim_output[9,1] <- mean(((sim_lnOR_1[,2]-lnOR_1)^2)^0.5)*(sim_size)^0.5
sim_output[10,1] <- mean(((sim_lnOR_1[,3]-lnOR_1)^2)^0.5)*(sim_size)^0.5
sim_output[11,1] <- mean(((sim_lnOR_1[,4]-lnOR_1)^2)^0.5)*(sim_size)^0.5
sim_output[12,1] <- mean(((sim_lnOR_1[,5]-lnOR_1)^2)^0.5)*(sim_size)^0.5
sim_output[13,1] <- mean(((sim_lnOR_1[,6]-lnOR_1)^2)^0.5)*(sim_size)^0.5
sim_output[14,1] <- mean(((sim_lnOR_1[,7]-lnOR_1)^2)^0.5)*(sim_size)^0.5
```

```
sim_output[15,1] <- mean(((sim_lnOR_2[,1]-lnOR_2)^2)^0.5)*(sim_size)^0.5
sim_output[16,1] <- mean(((sim_lnOR_2[,2]-lnOR_2)^2)^0.5)*(sim_size)^0.5
sim_output[17,1] <- mean(((sim_lnOR_2[,3]-lnOR_2)^2)^0.5)*(sim_size)^0.5
sim_output[18,1] <- mean(((sim_lnOR_2[,4]-lnOR_2)^2)^0.5)*(sim_size)^0.5
sim_output[19,1] <- mean(((sim_lnOR_2[,5]-lnOR_2)^2)^0.5)*(sim_size)^0.5
sim_output[20,1] <- mean(((sim_lnOR_2[,6]-lnOR_2)^2)^0.5)*(sim_size)^0.5
sim_output[21,1] <- mean(((sim_lnOR_2[,7]-lnOR_2)^2)^0.5)*(sim_size)^0.5

sim_output[22,1] <- mean(((sim_lnOR_3[,1]-lnOR_3)^2)^0.5)*(sim_size)^0.5
sim_output[23,1] <- mean(((sim_lnOR_3[,2]-lnOR_3)^2)^0.5)*(sim_size)^0.5
sim_output[24,1] <- mean(((sim_lnOR_3[,3]-lnOR_3)^2)^0.5)*(sim_size)^0.5
sim_output[25,1] <- mean(((sim_lnOR_3[,4]-lnOR_3)^2)^0.5)*(sim_size)^0.5
sim_output[26,1] <- mean(((sim_lnOR_3[,5]-lnOR_3)^2)^0.5)*(sim_size)^0.5
sim_output[27,1] <- mean(((sim_lnOR_3[,6]-lnOR_3)^2)^0.5)*(sim_size)^0.5
sim_output[28,1] <- mean(((sim_lnOR_3[,7]-lnOR_3)^2)^0.5)*(sim_size)^0.5

sim_output[29,1] <- mean(sim_mean_limit[,1])*(sim_size)^0.5
sim_output[30,1] <- mean(sim_mean_limit[,2])*(sim_size)^0.5
sim_output[31,1] <- mean(sim_mean_limit[,3])*(sim_size)^0.5
sim_output[32,1] <- mean(sim_mean_limit[,4])*(sim_size)^0.5
sim_output[33,1] <- mean(sim_mean_limit[,5])*(sim_size)^0.5
sim_output[34,1] <- mean(sim_mean_limit[,6])*(sim_size)^0.5
sim_output[35,1] <- mean(sim_mean_limit[,7])*(sim_size)^0.5
```

```
sim_output[36,1] <- mean(sim_lnOR_1[,1])*(sim_size)^0.5
sim_output[37,1] <- mean(sim_lnOR_1[,2])*(sim_size)^0.5
sim_output[38,1] <- mean(sim_lnOR_1[,3])*(sim_size)^0.5
sim_output[39,1] <- mean(sim_lnOR_1[,4])*(sim_size)^0.5
sim_output[40,1] <- mean(sim_lnOR_1[,5])*(sim_size)^0.5
sim_output[41,1] <- mean(sim_lnOR_1[,6])*(sim_size)^0.5
sim_output[42,1] <- mean(sim_lnOR_1[,7])*(sim_size)^0.5

sim_output[43,1] <- mean(sim_lnOR_2[,1])*(sim_size)^0.5
sim_output[44,1] <- mean(sim_lnOR_2[,2])*(sim_size)^0.5
sim_output[45,1] <- mean(sim_lnOR_2[,3])*(sim_size)^0.5
sim_output[46,1] <- mean(sim_lnOR_2[,4])*(sim_size)^0.5
sim_output[47,1] <- mean(sim_lnOR_2[,5])*(sim_size)^0.5
sim_output[48,1] <- mean(sim_lnOR_2[,6])*(sim_size)^0.5
sim_output[49,1] <- mean(sim_lnOR_2[,7])*(sim_size)^0.5

sim_output[50,1] <- mean(sim_lnOR_3[,1])*(sim_size)^0.5
sim_output[51,1] <- mean(sim_lnOR_3[,2])*(sim_size)^0.5
sim_output[52,1] <- mean(sim_lnOR_3[,3])*(sim_size)^0.5
sim_output[53,1] <- mean(sim_lnOR_3[,4])*(sim_size)^0.5
sim_output[54,1] <- mean(sim_lnOR_3[,5])*(sim_size)^0.5
sim_output[55,1] <- mean(sim_lnOR_3[,6])*(sim_size)^0.5
sim_output[56,1] <- mean(sim_lnOR_3[,7])*(sim_size)^0.5
```

```
sim_output[1,2] <- mean(((sim_mean_limit_biased[,1]-
exp(true_mean+sd_1^2/2))^2)^0.5)*(sim_size)^0.5

sim_output[2,2] <- mean(((sim_mean_limit_biased[,2]-
exp(true_mean+sd_1^2/2))^2)^0.5)*(sim_size)^0.5

sim_output[3,2] <- mean(((sim_mean_limit_biased[,3]-
exp(true_mean+sd_1^2/2))^2)^0.5)*(sim_size)^0.5

sim_output[4,2] <- mean(((sim_mean_limit_biased[,4]-
exp(true_mean+sd_1^2/2))^2)^0.5)*(sim_size)^0.5

sim_output[5,2] <- mean(((sim_mean_limit_biased[,5]-
exp(true_mean+sd_1^2/2))^2)^0.5)*(sim_size)^0.5

sim_output[6,2] <- mean(((sim_mean_limit_biased[,6]-
exp(true_mean+sd_1^2/2))^2)^0.5)*(sim_size)^0.5

sim_output[7,2] <- mean(((sim_mean_limit_biased[,7]-
exp(true_mean+sd_1^2/2))^2)^0.5)*(sim_size)^0.5


sim_output[8,2] <- mean(((sim_lnOR_1_biased[,1]-lnOR_1)^2)^0.5)*(sim_size)^0.5

sim_output[9,2] <- mean(((sim_lnOR_1_biased[,2]-lnOR_1)^2)^0.5)*(sim_size)^0.5

sim_output[10,2] <- mean(((sim_lnOR_1_biased[,3]-lnOR_1)^2)^0.5)*(sim_size)^0.5

sim_output[11,2] <- mean(((sim_lnOR_1_biased[,4]-lnOR_1)^2)^0.5)*(sim_size)^0.5

sim_output[12,2] <- mean(((sim_lnOR_1_biased[,5]-lnOR_1)^2)^0.5)*(sim_size)^0.5

sim_output[13,2] <- mean(((sim_lnOR_1_biased[,6]-lnOR_1)^2)^0.5)*(sim_size)^0.5

sim_output[14,2] <- mean(((sim_lnOR_1_biased[,7]-lnOR_1)^2)^0.5)*(sim_size)^0.5


sim_output[15,2] <- mean(((sim_lnOR_2_biased[,1]-lnOR_2)^2)^0.5)*(sim_size)^0.5

sim_output[16,2] <- mean(((sim_lnOR_2_biased[,2]-lnOR_2)^2)^0.5)*(sim_size)^0.5

sim_output[17,2] <- mean(((sim_lnOR_2_biased[,3]-lnOR_2)^2)^0.5)*(sim_size)^0.5

sim_output[18,2] <- mean(((sim_lnOR_2_biased[,4]-lnOR_2)^2)^0.5)*(sim_size)^0.5

sim_output[19,2] <- mean(((sim_lnOR_2_biased[,5]-lnOR_2)^2)^0.5)*(sim_size)^0.5
```

```
sim_output[20,2] <- mean(((sim_lnOR_2_biased[,6]-lnOR_2)^2)^0.5)*(sim_size)^0.5
sim_output[21,2] <- mean(((sim_lnOR_2_biased[,7]-lnOR_2)^2)^0.5)*(sim_size)^0.5

sim_output[22,2] <- mean(((sim_lnOR_3_biased[,1]-lnOR_3)^2)^0.5)*(sim_size)^0.5
sim_output[23,2] <- mean(((sim_lnOR_3_biased[,2]-lnOR_3)^2)^0.5)*(sim_size)^0.5
sim_output[24,2] <- mean(((sim_lnOR_3_biased[,3]-lnOR_3)^2)^0.5)*(sim_size)^0.5
sim_output[25,2] <- mean(((sim_lnOR_3_biased[,4]-lnOR_3)^2)^0.5)*(sim_size)^0.5
sim_output[26,2] <- mean(((sim_lnOR_3_biased[,5]-lnOR_3)^2)^0.5)*(sim_size)^0.5
sim_output[27,2] <- mean(((sim_lnOR_3_biased[,6]-lnOR_3)^2)^0.5)*(sim_size)^0.5
sim_output[28,2] <- mean(((sim_lnOR_3_biased[,7]-lnOR_3)^2)^0.5)*(sim_size)^0.5

sim_output[29,2] <- mean(sim_mean_limit_biased[,1])*(sim_size)^0.5
sim_output[30,2] <- mean(sim_mean_limit_biased[,2])*(sim_size)^0.5
sim_output[31,2] <- mean(sim_mean_limit_biased[,3])*(sim_size)^0.5
sim_output[32,2] <- mean(sim_mean_limit_biased[,4])*(sim_size)^0.5
sim_output[33,2] <- mean(sim_mean_limit_biased[,5])*(sim_size)^0.5
sim_output[34,2] <- mean(sim_mean_limit_biased[,6])*(sim_size)^0.5
sim_output[35,2] <- mean(sim_mean_limit_biased[,7])*(sim_size)^0.5

sim_output[36,2] <- mean(sim_lnOR_1_biased[,1])*(sim_size)^0.5
sim_output[37,2] <- mean(sim_lnOR_1_biased[,2])*(sim_size)^0.5
sim_output[38,2] <- mean(sim_lnOR_1_biased[,3])*(sim_size)^0.5
sim_output[39,2] <- mean(sim_lnOR_1_biased[,4])*(sim_size)^0.5
sim_output[40,2] <- mean(sim_lnOR_1_biased[,5])*(sim_size)^0.5
sim_output[41,2] <- mean(sim_lnOR_1_biased[,6])*(sim_size)^0.5
```

```
sim_output[42,2] <- mean(sim_lnOR_1_biased[,7])*(sim_size)^0.5


sim_output[43,2] <- mean(sim_lnOR_2_biased[,1])*(sim_size)^0.5
sim_output[44,2] <- mean(sim_lnOR_2_biased[,2])*(sim_size)^0.5
sim_output[45,2] <- mean(sim_lnOR_2_biased[,3])*(sim_size)^0.5
sim_output[46,2] <- mean(sim_lnOR_2_biased[,4])*(sim_size)^0.5
sim_output[47,2] <- mean(sim_lnOR_2_biased[,5])*(sim_size)^0.5
sim_output[48,2] <- mean(sim_lnOR_2_biased[,6])*(sim_size)^0.5
sim_output[49,2] <- mean(sim_lnOR_2_biased[,7])*(sim_size)^0.5


sim_output[50,2] <- mean(sim_lnOR_3_biased[,1])*(sim_size)^0.5
sim_output[51,2] <- mean(sim_lnOR_3_biased[,2])*(sim_size)^0.5
sim_output[52,2] <- mean(sim_lnOR_3_biased[,3])*(sim_size)^0.5
sim_output[53,2] <- mean(sim_lnOR_3_biased[,4])*(sim_size)^0.5
sim_output[54,2] <- mean(sim_lnOR_3_biased[,5])*(sim_size)^0.5
sim_output[55,2] <- mean(sim_lnOR_3_biased[,6])*(sim_size)^0.5
sim_output[56,2] <- mean(sim_lnOR_3_biased[,7])*(sim_size)^0.5
```
